# Measuring Socioeconomic Inequality Changes in Child Mortality in Iran: Two National Surveys Inequality Analysis

**Published:** 2018-09

**Authors:** Mostafa AMINI RARANI, Arash RASHIDIAN, Mohammad ARAB, Ardeshir KHOSRAVI, Ezatollah ABBASIAN

**Affiliations:** 1. Dept. of Health Management and Economics, School of Public Health, Tehran University of Medical Sciences, Tehran, Iran; 2. Health Management and Economics Research Center, Isfahan University of Medical Sciences, Isfahan, Iran; 3. Deputy of Public Health, Ministry of Health and Medical Education, Tehran, Iran; 4. Dept. of Economics, Bu-Ali Sina University, Hamadan, Iran

**Keywords:** Child mortality, Health disparities, Socioeconomic factors, Odds ratio, Iran

## Abstract

**Background:**

We aimed to measure changes in socioeconomic inequality in child mortality in Iran.

**Methods:**

A secondary data analysis of two Demographic and Health Surveys (DHS 2000 and 2010) was undertaken. Neonatal, infant and under-5 mortality rates were estimated directly from complete birth history. Economic quintiles were constructed using principal component analysis. Changes in inequality were measured using odds ratios, mortality rates, and concentration curves and indices.

**Results:**

Based on the compared measures, inequalities in neonatal, infant, and under-5 mortality declined between the two surveys. The poorest-to-richest neonatal, infant and under-5 mortality odds ratios in 2000 were 1.69 (95% CI= 1.3–2.07), 2.85 (95% CI= 1.96–4.1) and 1.98 (95% CI= 1.64–2.3), respectively. Whereas these mortality odds ratios in 2010 had fallen to 1.65 (95% CI= 0.95–2.9), 1.47 (95% CI=0.5–4) and 1.85 (95% CI=1.13–3), respectively. Moreover, mortality rates in all economic quintiles experienced a decreasing trend. Neonatal, infant, and under-5 mortality concentration indices in 2000 were −0.15, −0.26, and −0.17 respectively. Whereas concentration indices in 2010 had dropped to −0.13, −0.11, and −0.14, respectively. Concentration curves dominance test revealed that there was a statistically significant reduction in inequality in infant and under-5 mortalities.

**Conclusion:**

Despite substantial reduction in child mortality rates and narrowing of the gap between poor and rich people, socioeconomic inequality in child mortalities disfavoring worse-off groups still exists. Combination of child health-related efforts that aim to reach to those children born in poor households alongside with pro-equity programs in other sectors of society may further reduce infant, under-5, and particularly neonatal mortality across economic quintiles in Iran.

## Introduction

Health inequalities have become one of the prominent issues in policy agendas (1). In addition to achieving the desired average indices in relation to country’s health performance, distribution of health in the population is a key priority as well. Tackling health inequalities in mortality within countries has become an important objective of national governments and international organizations (2).

There are major and persistent inequalities between and within countries in child mortality that are less understood, especially in low and middle-income countries. Monitoring socioeconomic inequality in child mortality across countries and over time can demonstrate patterns and inconsistencies across different groups, and serve as a valuable way to examine why inequalities are large in some population than in others (3). Over the last two decades, most countries have managed, with millennium development goal 4 (MDG4), to decrease under-five yr mortality by 47% (4). Despite this, inequalities in child mortality between the poor and rich (5) or among different socioeconomic positions (6) continue to exist and even may have widened in recent years, especially in developing countries (2, 7). At the same time and paradoxically, inequalities in child mortality have decreased in recent years (8–11). However, because of such paradox and Millennium Development Goals mere attention to average levels of health status, one unanswered question is whether or not success in reduction of average child mortality rates have accompanied with reduction in inequalities in death rates between the poor and the non-poor groups. This concern, moreover, is in line with global demand for monitoring equity in health outcomes (12, 13) and notably, reducing inequality has been adopted as the tenth goal of the Sustainable Development Goals (SDGs) (14).

In Iran, trends indicate that substantial progress has been made in child health. Under-five mortality, infant mortality, and neonatal mortality rates in 1990 compared to 2013 declined from 57 to 17, 44 to 14, and 27 to 10, respectively (15). Furthermore, measuring socioeconomic inequality in maternal and children’s health outcomes in Iran indicated the persistence of inequality for all outcomes of interest (16). Moreover, inequality in healthcare expenditure has increased between the poor and non-poor over the past three decades and inflation has affected the poor more than the rich (17).

However, these trends also raise the question of how such inequalities have affected child mortality distribution, i.e. more concerning is the inequality in child mortality rather than average child mortality. Iran has been successful in meeting the MDG 4 (18), but children with high risk of mortality or children who live in disadvantaged households have lagged behind and advantaged populations benefitted from modern healthcare services and new child health interventions. If this is a case then the “inverse equity hypothesis” proposed by Victora et al may be observed, whereby reductions in overall child mortality rates mask increasing inequality (19).

Therefore, for the first time in Iran, we’ve measured inequality changes in neonatal, infant, and under-five mortality across wealth quintiles in 2000–2010, using absolute difference in child mortality odds ratios, mortality rates, concentration indices, and concentration curve dominance test.

## Materials and Methods

### Data

Data from Demographic and Health Survey (DHS) in 2000 (20) and Iran’s Multiple Indicator Demographic and Health Survey (IrMIDHS) in 2010 (21) were used. These data allow us a unique opportunity in measuring inequality due to the DHS 2000 and MIDHS 2010 were collected the rich nationally representative household’s data. The sample population in DHS 2000 included 2000 urban and 2000 rural households from 28 provinces of the country, plus 2000 households from the capital Tehran, totaling to 113957 households in overall. In IrMIDHS 2010 the minimum sample size was estimated to be 400 households in each province, totally the sample was about 31300 households.

Data from 113215 and 30870 households were analyzed to determine the economic status of Iranian households in 2000 and 2010, respectively. In addition, to measure inequality in child mortality, 45646 live births from DHS 2000 and 10604 live births from MIDHS 2010 were analyzed. All statistical analyses were performed in STATA 12/SE and Microsoft Excel 2010.

### Household economic status

An index of household economic status based on asset variables was constructed by using principal component analysis (PCA) (22, 23). More details with respect to the constructing households’ economic status can be found in the Amini Rarani et al.’s study (24).

### Child mortality estimates

Child death was a binary outcome variable, i.e. whether each of the live-born neonates (≤29 d), infants (≤11 months and 29 d), and children under-five years (≤59 months and 29 d) of the women interviewed was still alive or not. Three indices of child mortality were also estimated, namely neonatal mortality rate (NMR), infant mortality rate (IMR), and under-5 mortality rate (U5MR), defined respectively as all deaths from birth to 29 d of life, all deaths up to and including 1 year, and all deaths up to 5 yr. All child mortality rates are expressed as deaths per 1000 live births. Child mortality was directly estimated from complete birth history (CBH) based on a life-table approach and using the ‘ltable’ command in STATA 12/SE (22). Owning to relative scarcity of death, one-year death estimates of mortality are not adequately precise (7) and do not ensure enough births to reduce the effects of sampling error (5); hence, mortality rates were estimated by calculating the survival status of children using a 5-year observation period prior to the surveys extracted from birth histories of interviewed women. Namely, asking about women’s birth histories allow us to estimate survival status of children belonging to each interviewed women from years preceding the years of DHSs 2000 and 2010.

### Measurement of socioeconomic inequality

To measure socioeconomic inequality in child mortality a concentration index (CI) approach (25) was applied. This approach has been widely used to quantify and compare the degree of socioeconomic inequalities in health variables (5, 26). The CI is directly related to concentration curve (CC). If everyone regardless of economic status has precisely the same value of the health variable, the CC curve will be a 45-degree line called the “line of equality”. In contrast, if the health variable takes higher (lower) values among poorer people, the CC will lie above (blew) the line of equality. The CI is equaled to twice the area between CC and 45-degree line and its values can vary between −1 and +1 (22). The CI indicates the relationship between health variable and economic status, its sign shows the direction of the relationship and its magnitude reflects both the strength of relationship and degree of variability in the health variable distribution.

### Concentration curve dominance test

Concentration curve dominance test (22) was used to infer whether the change of CC in 2000 compared to CC in 2010 was statistically significant or not. To perform a dominance test two choices should be made: first, a decision (rule) about presence or absence of dominance, and second, a decision about number of quantile points at which ordinates are to be compared. Decision rule about dominance was made based on rejection of the null of non-dominance in favor of dominance if there was at least one significant difference between curves in one direction and no significant difference in another direction. One problem with such a decision rule is over rejection of the null (null of dominance) because there is no correction for the fact that multiple comparisons are being made. For solution, “multiple comparison approaches” was applied (27). Nineteen evenly spaced quantile points from 0.05 to 0.95 were selected, namely 0.95/0.05=19 (28). Moreover, the null hypothesis was written as sameness of CCs of neonatal, infant, and under-five mortality in 2000 and 2010 in each of the 19 points. If the null hypothesis is rejected, the curves did not intersect at any point and one curve dominates the other.

In the analysis of DHS data, three main factors that arose from the sampling design including stratification, cluster sampling, and unequal selection probabilities were considered. Accordingly, weight and cluster options in STATA commands were used to adjust for unequal sampling probabilities and to get standard errors right.

### Ethical approval

The study was approved by the Tehran University of Medical Sciences Research Ethics Committee with ethical code No: 136890.

## Results

Table 1 shows the results from a logistic regression of neonatal, infant and under-five mortality across economic quintiles and also the absolute differences of odds ratios. Odds ratio for poorest quintile compared to that of richest one in 2000 and 2010 were 1.69 (95% CI= 1.30–2.07) and 1.65 (95% CI= 0.95–2.90); 2.85 (95% CI= 1.96–4.10) and 1.47 (95% CI=0.50–4.0); and 1.98 (95% CI= 1.64–2.30) and 1.85 (95% CI=1.13–3.0) for neonatal, infant, and under-5 mortality rates respectively. These odds ratios showed that child mortality risks were higher in the poorest quintile than the richest one.

**Table 1:** Estimated neonatal (NM), infant (IM) and under-5 (U5M) mortality odds ratio and its 95 % confidence interval across economic quintiles, Iran (2000 and 2010)

***Quintiles***	***Odds Ratio (95% Conf. Interval)***						***Absolute difference***		
	***NM***		***IM***		***U5M***		***NM***	***IM***	***U5M***
	***2000***	***2010***	***2000***	***2010***	***2000***	***2010***			
Poorest	1.69^b^ (1.30,2.07)	1.65^c^ (0.95,2.90)	2.85^b^ (1.96,4.10)	1.47^c^ (0.50, 4.00)	1.98^b^ (1.64,2.30)	1.85^c^ (1.13,3.00)	−2.4	−68	−6.5
Poorer	1.68^b^ (1.22,1.95)	1.54^c^ (0.95,2.90)	2.25^b^ (1.50,3.30)	1.11^c^ (0.37,3.20)	1.74^b^ (1.44,2.10)	1.60^c^ (0.98,2.60)	−9	−50	−8
Middle	1.29^c^ (1.10,1.60)	0.88 (0.46,1.66	1.40 (0.84,1.96)	1.30 (0.45,3.70)	1.30^c^ (1.06,1.60)	1.09 (0.64,1.80)	−31	−7	−16
Richer	1.1 (0.85,1.42)	0.97 (0.50,1.86)	1.05 (0.67,1.60)	0.88 (0.27,2.90)	1.1 (0.92,1.40)	0.98 (0.55,1.70)	−12	−16	−11
Richest ^a^	1	1	1	1	1	1			

Notes: (^a^) denotes reference group. (^b^) and (^c^) denote *P*<0.001 and *P*<0.05, respectively

According to significance of odds ratios, two quintiles (the poorest and the poorer) had significant effects on neonatal, infant, and under-5 mortalities (*P*-value <0.05). For example, the odds ratio of poorest quintile for under-5 mortalities in 2000 and 2010 indicated that with moving up from the poorest to the richest quintile, survival probability of under-5 had 1.98 (98%) and 1.85 (85 %) times increase, respectively.

The negative values of absolute differences of odds ratios illustrated that odds ratio for neonatal, infant, and under-5 mortalities in all wealth quintiles had declined between 2000 and 2010. Table 2 presents child mortality rates per 1000 live births and their changes by household’s economic quintiles. NMR, IMR, and U5MR across wealth quintiles indicated that the higher the economic status, the lower the child mortality rate. Absolute differences in mortality rates demonstrated that mortality rates for neonatal, infant and under-5 mortalities in all economic quintiles had decreased in 2000–2010.

**Table 2:** Estimated neonatal, infant and under-five mortality rates (NMR, IMR and U5MR) per 1000 live births across economic quintiles, Iran (2000 and 2010)

***Quintiles***	***NMR (95% CI)***		***IMR (95% CI)***		***U5MR (95% CI)***		***Absolute difference***		
	***2000***	***2010***	***2000***	***2010***	***2000***	***2010***	***NMR***	***IMR***	***U5MR***
Poorest	26.4 (23.6,29.5)	19.4 (14.5,26)	40(36.4,43)	25.1 (19.3,32)	50 (45.6,54.7)	30.7 (23.4,40)	−26.5	−37.2	−38.6
Poorer	24.8 (22,28)	20.7 (15.5,27)	36.5 (33,40)	25 (19.3,32.7)	43.6 (39.5,48.2)	29 (21.8,38)	−16.5	−31.5	−33.4
Middle	20.8 (18,28)	10.5 (6.3,15)	27.3 (24,31)	14.9 (10.4,21)	32.5 (28.7,36.7)	17 (12,24)	−49.5	−45.4	−47.7
Richer	17.9 (11.6,21)	9.7 (6.5,16.8)	23.3 (20,27)	14.4 (9.6,21)	29.2 (25.3,33.7)	15.4 (10,23)	−45.8	−38	−47.2
Richest	16.2 (13.3,19.6)	9.3 (5,16.8)	21.3 (18,25)	11.1 (6.5,19)	25.6 (21.5,30.5)	12.2 (7.2,20)	−42.6	−47.8	−52.3

**Fig. 1:**
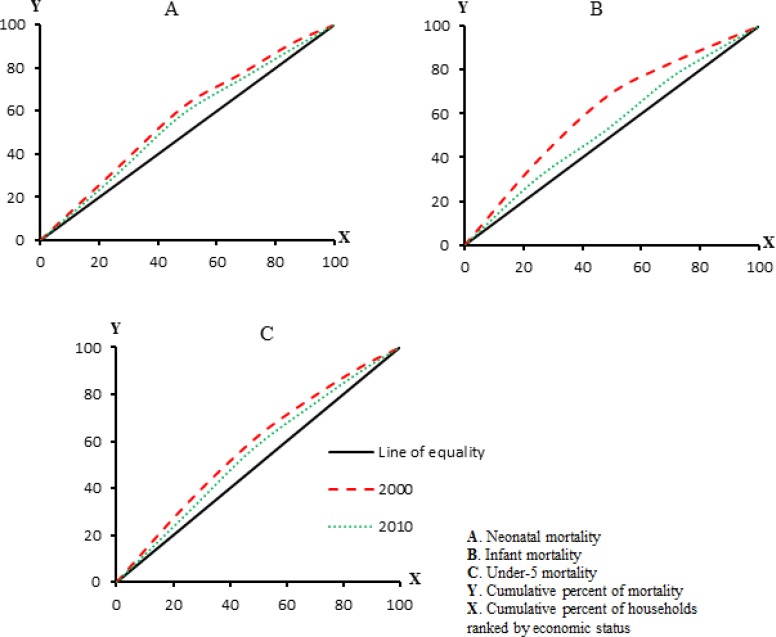
Concenteration curves of child mortality in Iran, (2000 and 2010)

Figure 1 illustrates the CCs for neonatal, infant, and under-5 mortalities. As the Fig. 1 (A) shows, concentration curves of neonatal mortality in 2000 and 2010 lied above the line of equality. Neonatal mortality concentrated more among the poor people. Moreover, CC in 2010 lied below the CC in 2000, implying that inequality in neonatal mortality had decreased in 2010. CI of neonatal mortality in 2000 was −0.15 (95% CI= −0.2 to −0.09), whereas it had dropped to −0.13(95% CI= −0.25 to −0.02) in 2010 a 13.3% reduction (from 15% to 13%). However, testing dominance of neonatal mortality CCs revealed that CC in 2000 did not dominate CC in 2010; that is, change of inequality in neonatal mortality between 2000 and 2010 was no statistically significant.

Fig. 1 (B) depicts CCs of infant mortality in 2000 and 2010, which lied above equality line. The CI of infant mortality in 2000 was −0.26 (95% CI= −0.34 to −0.18), while in 2010 it had decreased to −0.11 (95% CI= −0.29 to −0.06), a 57.7% reduction (from 26% to 11%).

The negative signs of CIs indicate that inequality in infant mortality was more concentrated across the worse-off households. Testing CCs dominance indicated that CC in 2000 dominated CC in 2010; that is, inequality change in infant mortality was statistically significant. Concentration curves for under-5 mortality are shown in Fig. 1(C). These curves indicated that inequality in under-5 mortality favored the better-off. The CI of under-5 mortality in 2000 was −0.17 (95% CI= −0.2 to −0.1), whereas it had dropped to −0.14 (95% CI= −0.22 to −0.05) in 2010 a 17.6% reduction (from 17% to 14%). Moreover, dominance test showed that CC in 2000 dominated CC in 2010, i.e. reduction in under-5 mortality inequality was statistically significant.

## Discussion

The neonatal, infant, and under-5 CCs and CIs indicated that child mortalities were unequally distributed across wealth quintiles in 2000 and 2010. In addition to successful reduction in the average level of NMR, IMR, and U5MR, Iran has experienced an improvement in reduction of child mortality inequalities. We found that the highest odds ratio, mortality rate, and CI reduction occurred in the poorest quintile of infant mortality, the richest quintiles of U5MR and infant mortality, respectively. In contrast, the lowest reductions observed in the odds ratio of the poorest quintile of neonatal mortality, poorer quintile of NMR and CI of neonatal mortality. Results from CC dominance test implied that inequality reductions in under-5 and infant mortality were statistically significant, but inequality change in neonatal mortality was not significant. The decrease in child mortality inequality implies that the scale-up of child health programs in Iran, mainly primary health care (PHC)-based programs, may have been successful in increasing access to child health care among underserved households, paving the way to achieve SDG 10 target regarding child health.

A study about inequality in IMR in Iran is consistent with our findings (5). Moreover, our findings are in line with evidence attained from other studies in different countries such as Rwanda (9), Chile (8), Thailand (11), Brazil (10), Cameroon, Nigeria, Malawi, Mozambique and Uganda (3). Nevertheless, some previous studies have reported increasing trends in socioeconomic inequality in child mortality (7, 29–32). However, in contrast, inequality in child mortalities have not, ostensibly, widened in Iran between 2000 and 2010. The findings of our study suggest that despite substantial reduction in child mortality rates and narrowing the gap between the poor and the rich in 2000–2010, socioeconomic inequality in child mortalities remains high in Iran. This might imply that wealthier households still more utilize modern services and new child health programs. According to “inverse equity hypothesis” (19), as a country scales up new health services and programs, it initially reaches to those with higher socioeconomic strata rather than to the poor, so early increase in inequality could be expected and only over time the gap between the poor and the rich will be improved. Our analyses imply that the hypothesis holds true in Iran because inequality in infant and under-5 mortalities has been narrowed significantly over a ten-year period. Nevertheless, neonates experienced the lowest reduction in the poorest-to-richest odds ratio, percentage of mortality rate, and concentration index. Neonatal mortality and inequality in neonatal mortality remains a challenge and needs to be more considered in child health policies.

Despite existence of inequality in child mortality in Iran, our results indicated that the child mortality gap between the poor and the rich households has reduced over time. This is mainly because of the set of pro-poor child health programs launched in Iran since 1979 in the form of PHC networks, health centers (in urban area), and health houses (in rural area) (33) improved accessibility of healthcare services for children. The PHC networks mostly target rural areas and include basic treatments, health education, maternal and child health services, family planning, immunization, and environmental health based on principles of health for all. Namely, these programs are usually scaled up for the poor, rural dwellers, and disadvantaged population through PHC. Specifically, some child health programs such as Integrated Management Child Illness, Well Baby Care program, and 1–59 months’ child mortality surveillance system have been implemented based on PHC principles in Iran over the last three decades. These PHC-based programs increase maternal and child health services accessibility to every district in the country, especially in rural and underprivileged areas resulted in more equitable allocation of health resources. Inequality in child mortality has seen a trend of reduction in Iran.

In addition, the family medicine program was developed and deployed in 2004 which expanded the healthcare services to urban and suburban areas (34). The family medicine program emphasizes the role of government to create logical and equitable public access to health services through rationing and implementation of basic health insurance on the basis of family medicine and referral path systems (35). This program has had some achievements in betterment of population healthcare and health insurance coverage (17). Such initiatives have helped health system in Iran to narrow child mortality inequality.

The present study had several limitations. First, as estimates were derived from cross-sectional data, it does not allow one to infer causality; hence, any attribution of causality should be made cautiously. Second, inaccurate estimation of inequality in child mortality, due to possible recall bias in two surveys, was different across economic quintiles. Third, the child mortality rates were derived from periods before the time of the DHS, but economic quintiles of households were from the year of the survey (time inconsistency limitation). However, the long run nature of the economic status indicator may help to lessen the effects of this issue. All these limitations should be borne in mind when reading and explaining the results. Nonetheless, there are some strengths to our study: a) use of DHS and MIDHS studies comprised of a rich set of nationally representative data and ensure the generalization of the results; and b) measurement of household economic status using indirect measure (household durable asset index) constructed by PCA method instead of direct measure (such as income or expenditure). Because of non-expensiveness, practical and somewhat unbiased approach, the PCA method, particularly in developing countries, has less limitation in comparison to direct measures.

## Conclusion

We showed a decline in the poorest-to-richest child mortality odds ratios, child mortality rates across wealth quintiles, and child mortality inequality in Iran between 2000 and 2010. Nevertheless, from health equality perspective, the gap between the poor and the rich in child mortality still exists, disfavoring the worst-off groups. In Iran, combination of child health-related efforts that aim to reach to those children born in poor households alongside with pro-equity programs in other sectors of society may further decrease infant, under-5, and particularly neonatal mortality.

## Ethical considerations

Ethical issues (Including plagiarism, informed consent, misconduct, data fabrication and/or falsification, double publication and/or submission, redundancy, etc.) have been completely observed by the authors.
